# Perampanel overdose in low body mass index patients with epilepsy: a case report and review of the literature

**DOI:** 10.1186/s13256-021-02759-9

**Published:** 2021-03-29

**Authors:** Kanitpong Phabphal, Prut Koonalintip

**Affiliations:** Neurology Unit, Department of Internal Medicine, Faculty of Medicine, Prince of Songkla University, Hat Yai, Songkhla, 90110 Thailand

**Keywords:** Perampanel, Overdose, Epilepsy

## Abstract

**Background:**

Perampanel (PER) is a novel antiepileptic drug (AED) which employs a completely different mechanism of action compared to existing medications. Overall, PER is considered to be safe up to a dose of 12 mg per day. When used to treat refractory and super-refractory status epilepticus, PER seems to be extremely well tolerated; this is true even when used at doses of up to 32 mg. There are currently only three case reports on the effects of acute PER overdose in epilepsy patients.

**Case presentation:**

We report a 16-year-old Thai woman with a low body weight, who took PER at a dose of 40 times that of the prescribed daily dose. She experienced only an alteration of consciousness, without any systemic medical effects, and made a full recovery within 3 days without gastric lavage or specific treatment.

**Conclusion:**

Our report demonstrates that an acute PER overdose may not produce serious adverse systemic effects. Individuals with adverse central nervous system (CNS) effects, such as altered consciousness, can experience a rapid recovery.

## Background

Among the currently available antiepileptic drugs (AEDs), perampanel (PER) is a novel AED with a completely different mechanism of action. It is the first selective, noncompetitive α-amino-3-hydroxy-5-methyl-4-isoxazolepropionic acid (AMPA) receptor antagonist used to treat epilepsy. Clinical trials have shown that PER is generally well tolerated, though dose-dependent central nervous system (CNS) effects occur. In the short term, the most commonly reported treatment-emergent adverse events (TEAEs) leading to dose reductions or treatment interruptions are dizziness, somnolence, headache, and fatigue [1]. PER, at 2 mg/day, has been demonstrated to have similar effects and side effects as those of a placebo [2]. At doses of 4 mg/day, dizziness, somnolence, and irritability have been reported as adverse events (AEs). TEAEs are generally more often reported at doses of 8 or 12 mg/day [3]. An extension study of a trial held over a period of 4 years revealed that the most common TEAEs leading to PER therapy discontinuation were dizziness (4.1%), irritability (1.2%), and fatigue (1.1%) [4]. The efficacy of perampanel has been evaluated extensively in various phase II, randomized placebo-controlled phase III [1, 2], and open-label extension trials [5]. The patients recruited in these studies were ≥12 years of age, had refractory partial seizures with or without secondary generalization, and were on 1–3 additional AEDs [2, 4, 6].

The usual starting dose of PER for focal and generalized seizures in adult patients who are not concomitantly taking an enzyme-inducing antiepileptic drug is 2 mg/day before sleep. Additionally, it is recommended that the dose be increased by 2 mg/day biweekly to reach the maximum tolerable dose (usually 4–8 mg/day), which provides excellent seizure control. Overall, PER is considered to be safe up to a daily dose of 12 mg per day. When used to treat refractory and super-refractory status epilepticus (SE), PER seems to be extremely well tolerated; the same holds true even when used at doses of up to 32 mg. However, currently, very little has been published about incidents where PER is taken above the recommended dosages [7–10]. Three patients with epilepsy [7–9] and one patient with depression and anxiety [10] have been reported to have experienced PER intoxication. Those reports included adult patients aged 34, 39, 40, and 54 years. None of the four studies on PER intoxication reported the body mass index (BMI) of the participants. However, all participants enrolled in the core study investigating the efficacy of PER had a normal BMI. With regard to the pharmacokinetics, PER undergoes extensive hepatic metabolism (>90%) to form 13 major metabolites of hydroxylated perampanel and various glucuronide conjugates. The isoenzyme CYP3A4 is considered to be primarily involved in the metabolism of PER [11]. In a recent study on the correlation between body weight and CYP3A activity, [12] Sandvik *et al.* reported that the enzyme activity of CYP3A4 was higher in patients with anorexia nervosa compared to a control group consisting of individuals with normal weight, and that CYP3A4 activity was negatively correlated with body weight [13]. Also, CYP3A4 activity was found to decrease with increasing body weight in healthy volunteers [14].

Herein, we describe the clinical findings in a low-BMI patient with epilepsy, who ingested an estimated 80 mg of PER in a suicide attempt. This study was approved by the institutional review board of the Faculty of Medicine, Prince of Songkla University (REC.62-396-14-3).

## Case presentation

The patient was a 16-year-old Thai female who had suffered from refractory epilepsy since the age of 10. She had a focal onset with impaired awareness and motor predominance at least five times per month. Magnetic resonance imaging studies revealed a non-enhancing diffuse enlargement of the right amygdala, hippocampus, and parahippocampal gyrus, and electroencephalography showed occasional spikes in the corresponding areas. She weighed 32 kg (BMI = 19). She had been on multiple antiepileptic drugs, which include valproate 2000 mg/day, levetiracetam 1500 mg/day, and lamotrigine 50 mg/day. At the age of 16, she experienced multiple attacks of convulsive SE, so she was transferred to our hospital for more specialized treatment. A mood disorder was detected on that visit, but no relevant mood conditions before or during treatment at our department were reported by either the patient or her family. Moreover, neither suicidal thoughts nor previous suicide attempts were reported. The patient was initially treated by increasing the lamotrigine dose to 100 mg/day, but her seizures persisted. Two milligrams per day of PER before sleep was added as adjunctive therapy; the dose was up-titrated to 4 mg/day after 2 weeks. Subsequently, she was seizure-free for 6 months. The seizures were controlled with combination therapy consisting of 4 mg/day of PER, the withdrawal of valproate and levetiracetam, and the tapering of lamotrigine to 25 mg/day. We then decided to switch to PER monotherapy by tapering off lamotrigine. The patient had been seizure-free for 8 months when she was found drowsy in her bedroom by her friends, with an empty bottle of PER; there were approximately 40 tablets missing (80 mg). On physical examination, her mental status was described as a state of confusion, and she exhibited signs of withdrawal in response to painful stimuli. Her Glasgow Coma Scale (GCS) score was 8. The other physical findings were as follows: normal vital signs; skin that felt warm to the touch; and pupils that were equal, round, and reactive to light. In addition, her reflexes were normal, there were no focal neurological signs or symptoms, and her mucous membranes were moist. Moreover, no tremors were noted, and her gag reflex was intact. No specific treatment was administered in the emergency room; specifically, no gastric lavage was performed because the time of ingestion was more than 3 hours prior to presentation. It is known that oral PER is rapidly and almost completely absorbed (time to maximum concentration [*T*_max_], 0.5–2.5 hours), is not subject to any significant first-pass metabolism, and has a bioavailability of almost 100%. Her initial laboratory results were normal, and comprised the findings of a complete blood count, electrolyte test, liver function test, and renal function test. A urine drug and toxic substance screen was negative for benzodiazepines, amphetamine, and ethanol. Additional testing including head computed tomography (CT), chest X-ray, and electrocardiography showed no acute abnormalities. An electroencephalogram performed on the day after admission demonstrated a diffuse slowing and disorganization of the background, consistent with generalized dysfunction. Unfortunately, we were unable to determine serum concentrations due to the unavailability of the technology to measure the blood levels of PER in Thailand.

During the patient’s hospital stay, her symptom of drowsiness changed to a qualitative impairment of consciousness involving disorientation and misjudgment of situations, which resolved in 2 days (full GCS score). Her vital signs and other laboratory results remained stable during the first 2 days of admission, and there was no evidence of perampanel-induced systemic AEs such as hepatic or renal toxicity, hypotension, or respiratory suppression. Throughout hospitalization, her vital signs remained within normal parameters. She was discharged on admission day 3 with a normal level of consciousness as well as other neurological and general medical parameters. Once medically cleared, she voluntarily agreed to transfer to the psychiatry ward. During her time there, she was diagnosed with severe depression induced by familial problems. During consultation with the attending staff there, she said, “It was just a blind rage; I did not mean to do that. I am so sorry.” The psychiatric condition leading to her suicide attempt was the diagnosed major depressive syndrome. She was put on an antidepressant, and her depressive symptoms partially resolved. She was deemed psychiatrically stable and was discharged with a prescription of sertraline 50 mg daily and outpatient psychiatric follow-up care. At the initial follow-up 2 weeks after discharge, she reported mood changes with depressive symptoms, which had begun after the tapering off of lamotrigine, and at the same time she was experiencing some family problems; she did not notify her doctor about this. Two months after discharge, she was taking lamotrigine 100 mg/day and sertraline 50 mg/day.

## Discussion

There is sparse information regarding the effects of PER in cases of an acute overdose. Hence, the definition of PER overdose is not clear. However, we defined it as having received more than 32 mg/day of PER. Herein, we report on a patient from Thailand with a low BMI who overdosed on PER 80 mg/day. Our patient was prescribed a relatively low dose (4 mg/day), so the dose she ingested (80 mg/day) was 20 times higher than the upper limit of her daily dose. The neurological signs in this case involved a significantly depressed mental status manifested by her symptom of drowsiness. Interestingly, her symptoms persisted for 2 days, after which a rapid and complete recovery of her clinical condition was observed. These findings are in contrast to those reported by Kim *et al*. involving a 39-year-old woman admitted due to altered consciousness and stupor after taking 10 times the daily dose of both PER and valproic acid (she had been taking 4 mg/day of PER and 2000 mg of valproate). In that case, the patient began to regain consciousness on hospitalization day 6 and returned to her baseline mental status on day 8 [7]. Likewise, Li *et al.* reported prolonged stupor in a 54-year-old man who had experienced PER intoxication [8].

Our patient ingested 20 times the prescribed PER dosage, which is double that reported by Kim *et al.* The reason why our patient recovered so quickly from altered consciousness may be the fact that she took only PER without any concomitant drugs, especially enzyme inhibitors, whereas the case reported by Kim *et al.* had taken PER and valproic acid (an enzyme inhibitor antiepileptic drug). There are at least three routes of valproate metabolism: glucuronidation (50%), β oxidation (40%) in the mitochondria, and cytochrome P450 (CYP)-mediated oxidation (10%) [15]—then did not effect PER metabolism. Kim *et al.* believed that the prolonged stupor in that case was due to the prolonged half-life of PER, not the result of valproate encephalopathy. Another possible explanation is the low BMI of our patient. A previous study reported a negative correlation between BMI and CYP3A4 levels. CYP3A4 is a major metabolite of PER, and an increase in the level of CYP3A4 results in an increase in PER metabolism. Nevertheless, a comprehensive understanding of this mechanism is lacking and requires further study. According to clinical trials, the most commonly reported AEs from therapeutic dosing are dizziness, somnolence, fatigue, and irritability [3, 4]. However, little information on the effects of PER overdose is available. Case reports on PER overdose have revealed that the most frequent clinical presentations involve altered consciousness (stupor) and severe aggression (Table 1) [7–10].

**Table 1:** Characteristics of perampanel overdose patients from different case reports

Case report	Age (years)	Previous diagnosis	Weight	Dose of perampanel	Co-medications	Vital signs	Initial neurological manifestation	Systemic manifestation	Duration to recovery	Specific treatment	Full recovery
Hoppner *et al.*	35	Refractory epilepsy	NA	204 mg (25.5 times her daily dose)	Levetiracetam Topiramate Pregabalin	NA	Dysarthria followed by stupor	No	2 days	No^a^	Yes^b^
Li *et al.*	54	Refractory epilepsy	NA	300 mg	Divalproex sodium Lamotrigine Levetiracetam	Heart rate 58/minute Other findings were unremarkable	Marked somnolence Disconjugate gaze with 3 mm reactive pupils bilaterally	Serum sodium 128 mEq/L	4 days	No^c^	Lost to follow-up
Kim *et al.*	39	Epilepsy	NA	40 mg (10 times her daily dose)	Valproic acid	Normal	Stupor	Normal^d^	8 days^e^	No^c^	Yes^b^
Wu *et al.*	40	Depression and anxiety disorders, polysubstance abuse	NA	60 mg^f^	NA	Respiratory rate 6/minute^g^	Unresponsive then extremely agitated next day	Normal	5 days	No^c^	NA
Our report		Refractory epilepsy		80 mg (40 times her daily dose)	Lamotrigine	Normal	Drowsiness	Normal	3 days	No^a^	Yes^b^

*NA* not available

^a^Involved gastric lavage

^b^Full recovery to baseline before perampanel overdose

^c^No report of gastric lavage

^d^Pulmonary embolism on day 3

^e^Duration to recovery comprised the time required for recovery from both perampanel overdose and pulmonary embolism

^f^First time of perampanel ingestion

^g^Urine screening positive for benzodiazepine and ethanol

With respect to the systemic AEs of PER, data related to vital signs, clinical laboratory parameters, and electrocardiographic findings [1, 2, 16] have revealed that patients taking PER in combination therapy are more likely to experience AEs than those on PER monotherapy. Additionally, a dose–response relationship was discovered. Overall, most reported TEAEs were considered mild or moderate in severity; no deaths were recorded, but severe TEAEs were reported by 22 patients (5.0%) receiving placebo and 57 (5.5%) receiving PER at any dose. The serious TEAEs included falls, nephrolithiasis, cholelithiasis, hemorrhagic cystitis, urinary incontinence, and thrombocytopenia. There have been no reports of unstable vital signs, liver toxicity, abnormal complete blood count findings, or cardiac conduction defects. In terms of PER overdose cases, Wu *et al.* reported a psychiatric patient with a PER overdose and a urine screen that was positive for benzodiazepine and ethanol. They described a patient with severe respiratory depression, who was unresponsive. He was intubated due to concerns related to his airway protection [10]. Another report by Li *et al.* involving epileptic patients with PER overdose described findings of asymptomatic bradycardia and hyponatremia with spontaneous recovery without specific treatment [8]. Our patient reportedly ingested up to 80 mg of PER (40 times her daily dose) and experienced a short period of drowsiness with a significantly depressed mental status and minimal reaction to painful stimuli while maintaining her airway reflexes, which did not warrant endotracheal intubation. In addition, our report points out that an acute PER overdose may not produce serious adverse systemic effects such as cardiac toxicity, respiratory depression, or other metabolic derangement symptoms. This finding is supported by the results reported by Hopper *et al.* [9] and Kim *et al.* [7], which involved patients that had intentionally overdosed on PER as a suicide attempt and experienced symptoms such as impaired consciousness and agitation, without any significant systemic AEs.

As of now, there is no specific treatment for PER overdose. With regard to its pharmacokinetic properties, the PER concentration has been found to peak after 60 minutes. In phase I studies among healthy male volunteers, the mean plasma half-life of PER was found to range from 52 to 129 hours following a single-dose administration; this could explain our patient’s short duration of coma. If patients present to the hospital more than 60 minutes after ingestion, gastric lavage may not be necessary. According to previous single-case reports, patients recover 2 to 5 days after admission [7–10] without gastric lavage or specific treatment. However, the plasma half-life of PER may be shorter or longer than that of a single dose of PER in the presence of co-medication with cytochrome P450 enzyme inducers or inhibitors.

## Conclusion

Our report demonstrates that an acute PER overdose may not produce serious adverse systemic effects such as cardiac toxicity, respiratory depression, or other metabolic derangement symptoms. In addition, adverse CNS side effects such as altered consciousness may experience rapid recovery.

## Data Availability

Not applicable.

## Abbreviations

AEDs: Antiepileptic drugs
AMPA: α-Amino-3-hydroxy-5-methyl-4-isoxazolepropionic acid
PER: Perampanel
SE: Status epilepticus
